# *γ*-Aminobutyric acid (GABA) production and soluble free amino acid profile change in Andean seeds by *Levilactobacillus brevis* fermentation

**DOI:** 10.3389/fnut.2025.1693053

**Published:** 2025-11-07

**Authors:** Gabriela Ibieta, Jimena Ortiz-Sempértegui, Carl Grey, J. Mauricio Peñarrieta, Javier A. Linares-Pastén

**Affiliations:** 1 Biotechnology and Applied Microbiology, Faculty of Engineering LTH, Lund University, Lund, Sweden; 2 Instituto de Investigaciones Químicas IIQ, Universidad Mayor de San Andrés UMSA, La Paz, Bolivia

**Keywords:** solid state fermentation (SSF), *Levilactobacillus brevis*, *γ*-aminobutyric acid (GABA), amino acid profile, Andean seeds, short-chain fatty acids (SCFAs)

## Abstract

High nutritional value Andean seeds—tarwi (*Lupinus mutabilis*), cañihua (*Chenopodium pallidicaule*), and quinoa (*Chenopodium quinoa*)—were subjected to solid-state fermentation with *Levilactobacillus brevis* DSM 1269. This strain can convert L-glutamic acid into the neurotransmitter GABA. Fermented tarwi exhibited the highest GABA production, at 4 mg/g sample, which correlates with its higher protein content compared to fermented quinoa and cañihua, at 1 mg/g and 0.3 mg/g, respectively. Seeds kept at room temperature before fermentation produced higher concentrations of GABA compared to seeds kept at 4 °C. Autoclaving, a mandatory step for fermentation, resulted in a decrease in L-glutamic acid in tarwi seeds and an increase in quinoa and cañihua seeds. Additionally, fermentation produced lactic acid and acetic acid, together with an increase in the content of free essential amino acids, including threonine, histidine, methionine, isoleucine, leucine, valine, and lysine. This work demonstrated, for the first time, the functional valorisation of tarwi, cañihua, and quinoa through the production of bioactive metabolites and the enhancement of essential free amino acids via fermentation with *L. brevis*.

## 1 Introduction

Fermentation is an ancient process originally developed to extend the shelf life and improve the sensory properties of food (1). Today, fermentation is a biotechnological tool used for developing new food products with enhanced nutritional and functional properties (2). Lactic acid bacteria (LAB) strains are of interest in food matrices fermentation because they can improve the physicochemical and sensory properties, also can produce enzymes and bioactive metabolites such as peptides, exopolysaccharides, bacteriocins, vitamins, short-chain fatty acids (SCFAs), γ-amino butyric acid (GABA), and other metabolites (3–5). Additionally, many LAB strains are considered probiotics, conferring beneficial effects on human health.

Fermentation also affects the proteins and amino acid profile (6). It is particularly interesting for improving the nutritional and functional value of plant proteins. Some LAB strains express proteases that can release peptides and free amino acids from the food matrices, increasing the content of free amino acids. On the other hand, the content of free amino acids can also decrease if the strain utilises them in its own metabolism (7). Therefore, quantifying the free amino acid profile before and after fermentation provides insights into the microorganism’s activity towards proteins. A desirable amino acid profile can include the increment of essential amino acids or precursors of bioactive metabolites, for example, tryptophan that later can be converted into serotonin, or glutamic acid, which can be converted into GABA.

GABA is a non-protein amino acid that has gained interest due to its numerous health benefits for humans (8). GABA is the primary inhibitory neurotransmitter in the mature central nervous system (9). It has also been proven to prevent and treat diseases, including diabetes, depression, anxiety, and chronic insomnia (10–13). Another important property of GABA is its role as a mediator in the gut–brain axis (14).

*Levilactobacillus brevis* has been studied by many authors as an efficient GABA-producing strain. It produces GABA primarily as a mechanism to tolerate and survive acidic conditions within its environment (15). For example, *L. brevis* strains isolated from Chinese pickles (LBG-29 and LBG-24) could be utilised to enhance beverage functionality due to their high GABA production (>300 mg/L) (8). Another study demostrated the production of GABA in soybean via fermentation with *Levilactobacillus brevis* NPS-QW 145, resulting in 2.3 g/L of GABA, which can be used to produce a yoghurt-like product with enhanced GABA levels (16). Studies have also shown that *L. brevis* CRL 2013 does not possess antibiotic resistance genes and virulence markers in its genome, making it safe for probiotic applications (17).

Seeds, such as beans, rice, and sesame, are natural sources of GABA (18). However, in seeds with low or undetectable GABA levels, its content can be increased through two primary approaches: germination or fermentation. In this study, three Andean seeds with undetectable GABA levels —tarwi (*Lupinus mutabilis*), cañihua (*Chenopodium pallidicaule*), and quinoa (*Chenopodium quinoa*) — were selected for GABA enhancement via fermentation with *L. brevis*. These seeds are attractive due to their high nutritional value and sustainable production conditions, including high protein content and diverse amino acid profile (19, 20). For these seeds, very little literature is found; for example, quinoa was fermented with *L. plantarum* and subjected to an additional cold stress treatment, which improved GABA production (21). In comparison, cañihua and tarwi have only been reported to produce GABA by germination (22).

Solid-state fermentation (SSF) is considered a sustainable biotechnology that utilises low amounts of water to transform solid plant-based substrates into bioactive compounds (23). SSF is well-known in the food industry as a method for producing metabolites, enzymes, and organoleptic properties such as flavour and colour (24). SSF has also been studied for GABA production; for example, a recent study showed that SSF of hemp by *Lactobacillus casei* and *Bacillus subtilis* can produce a GABA-enhanced drink, yielding a product with 102.45 mg/100 mL of GABA, compared to 79.84 mg/100 mL of GABA produced by germination (25). Recently, GABA was produced in tarwi, cañihua, and quinoa Real seeds by enzymatic treatment with *L. brevis* glutamate decarboxylase (GAD), GadA, and GadB, where the highest yield was obtained in tarwi seeds by GadB; 2.46 mg/g of dry sample in 1 mL reactions (26).

In this context, the production of GABA by fermentation of tarwi, cañihua, and quinoa with *L. brevis* is reported for the first time. Alongside GABA, short-chain fatty acids (SCFAs) were also quantified as an indication of fermentation extent. Additionally, the quantification of soluble free amino acids was performed both before and after fermentation, considering the autoclavation step.

## 2 Materials and methods

### 2.1 Chemicals

De Man, Rogosa and Sharpe (MRS) Broth media, MRS agar, NaClO, L-glutamic acid, *γ*-aminobutyric acid (GABA), dansyl chloride, acetic acid, sulphuric acid, sodium phosphate dibasic heptahydrate, sodium phosphate monobasic monohydrate, ammonium acetate (LC–MS and HPLC grade), acetonitrile (LC–MS and HPLC grade), short-chain fatty acids standards; acetic acid, formic acid, propionic acid and butyric acid, lactic acid, amino acid standard AAS18 (Supelco). All reagents were purchased from Sigma Aldrich at analytical grade.

### 2.2 Bacterial strain

*Levilactobacillus brevis* DSM 1269 was purchased from Leibniz Institute DSMZ—German Collection of Microorganisms and Cell Cultures GmbH.

### 2.3 Bacterial strain stock preparation

The MRS broth was prepared in 51 g/L distilled water according to the manufacturer (Sigma-Aldrich). Subsequently, 25 mL of the medium was dispensed into a 40 mL Wheaton serum bottle. The medium was then deprived of oxygen by bubbling it with N_2_ for 5 min, and the bottle was closed with rubber stoppers. The bottle was sterilised in an autoclave.

Under sterile conditions, 1 mL of the medium was transferred from the Wheaton serum bottle to a 1.5 mL Eppendorf tube. A freeze-dried bacterium strain was resuspended in the medium and then transferred to the Wheaton serum bottle using another syringe. Following the recommendations for handling freeze-dried bacterial strains from Leibniz Institute DSMZ—German Collection of Microorganisms and Cell Cultures GmbH.

The bacterium was incubated at 30 °C for 24 h. Then, using a sterile syringe on the sterile bench, 100 μL of a bacterial sample was taken for cultivation on an agar plate. The plate was incubated at 30 °C for 24 h in an anaerobic chamber. Several colonies were observed on the plate and kept at 4 °C.

### 2.4 Seeds sampling

Cañihua (*Chenopodium pallidicaule*) seeds were collected from Chojñacota, Gualberto Villarroel Province (coordinates: 17°29′41.469″S 68°03′53.368″W), with an altitude of 3,770 (m.a.s.l.). Tarwi (*Lupinus mutabilis*) seeds were collected from Carabuco—Puerto Mayor de Carabuco, Camacho Province (coordinates: 15°44′00″S 69°01′00″W), with an altitude of 3,904 (m.a.s.l.). Both collection sites are within the Department of La Paz, Bolivia. Finally, a sample of commercial quinoa available in Swedish supermarkets was added to the study.

### 2.5 Seeds disinfection

Before disinfection, the moisture of the seeds was determined in triplicate, using a moisture analyser (Radwag MAC 110/WH, Poland).

For disinfection, 20 g of each seed was washed with 60 mL of 1% NaClO (v/v) for 30 min. It was washed three times with ultra-pure water (Milli-Q) and left overnight in 200 mL of Milli-Q water. The next day, the seeds were rewashed twice with Milli-Q water.

The seeds were then divided into two groups: one kept at room temperature (approximately 20 °C) for 24 h and the second at 4 °C for 24 h. Then, both groups were freeze-dried and ground into flour. The samples were named cañihua treated at 4 °C (C-4), cañihua treated at room temperature (C-RT), and the same for tarwi and quinoa: T-4, T-RT, Q-4, and Q-RT, respectively. These prior treatments of the seeds were tested because a previous work determined that a cold stress treatment on the seeds enhances GABA production through fermentation of quinoa seeds (21).

### 2.6 Fermentation

Each fermentation was conducted in duplicate due to the limited availability of raw samples and the high analytical demand of LC–MS/HPLC quantification. However, we highlight that all analytical measurements were performed in duplicate for each replicate, and the results showed high consistency between duplicates, indicating good reproducibility.

The fermentation setting consisted of using 2 g of each previously freeze-dried sample in Wheaton serum bottles with 25 mL of Milli-Q water. Then, the bottles were deprived of oxygen by bubbling them with N_2_ for 5 min and then closed with rubber stoppers. The bottles were sterilised in an autoclave.

Then, 200 μL of the bacterial stock (*L. brevis*, 25 CFU/μL) was introduced to the sterile bottles. The fermentation took place for 48 h at 30 °C. These parameters were determined as optimal for GABA production by *L. brevis* fermentation in the literature (21, 27).

To determine the impact of autoclaving on the conversion of L-glutamic acid into GABA, controls were performed using the same amounts, both with and without autoclaving. Then, both control groups were left for 48 h at 30 °C.

Given that solid-state fermentation was performed, the OD does not directly demonstrate the bacterial growth. It can demonstrate the presence of particles in solution (metabolites), which are products of fermentation. Therefore, the fermentation was determined by quantifying SCFAs, lactic acid, and GABA, which were measured in the aqueous fraction.

### 2.7 Derivatisation of amino acids and GABA for HPLC analysis

To quantify free amino acids and GABA by HPLC, performing derivatization before analysis is essential. The method was used previously in the group (26). In Eppendorf tubes, an aliquot of 150 μL of each sample was taken. Then, 300 μL of 0.1 M sodium bicarbonate buffer (pH 9.8), 150 μL of 10 mg/mL dansyl chloride in acetonitrile, and 900 μL of water were added. The tubes were incubated for 40 min at 80 °C. Finally, 150 μL of 20 μL/mL acetic acid was added to stop the reaction, and the tubes were centrifuged for 5 min at 12,000 rpm.

The supernatants were filtered through 0.2 μm pore size polytetrafluoroethylene (PTFE) membrane filters and stored at −20 °C until analysis by LC–MS and HPLC.

### 2.8 Identification of amino acids and GABA by liquid chromatography-mass spectrometry (LC–MS)

A liquid chromatography-mass spectrometry system, Dionex Ultimate 3,000 UHPLC (Thermo Fisher Scientific, Rockford, Illinois, United States), LTQ-Velos pro mass spectrometer using ESI in positive mode, connected to a UV detector at a wavelength of 275 nm equipped with a Kinetex 2.6 μm EVO C18 100 Å column 100 × 2.1 mm (Phenomenex, Torrance, California, United States), was used to identify amino acids and GABA. Two mobile phases were used: 30 mM ammonium acetate (A) and acetonitrile (B). A gradient between the solvents was employed; the separation starts with 18% of B. After 13 min, it increases to 22%, then when the total time reaches 16.5 min, B increases to 16.5%, and at a total time of 20.5 min, B increases to 30%. At a total time of 24 min, B decreases to 18%, and finally, at a total time of 27 min, B decreases to 10%. The method was based on a previous work (26) with modifications. Ten microliters of the sample were injected, the flow was maintained at 0.3 mL/min during the analysis, and the column temperature was set at 30 °C.

### 2.9 Quantification of amino acids and GABA with HPLC

After the identification of all amino acids and GABA by LC–MS, their quantification was performed using the same method described in section 2.8 in a high-performance liquid chromatography (HPLC) Vanquish (Thermo Fisher Scientific, Waltham, Massachusetts, United States) system connected to a UV detector at a wavelength of 275 nm equipped with the Kinetex 2.6 μm EVO C18 100 Å column 100 × 2.1 mm (Phenomenex, Torrance, California, United States).

### 2.10 Statistical analysis of amino acid profile and GABA production

Principal component analysis (PCA), heat maps, and dendrograms were performed on the soluble amino acid profiles of samples, including control groups and fermented samples, using Python (version 3.11) within a Jupyter Notebook environment. Cohen’s d test was applied to GABA production in fermented samples. Visualizations of the PCA, heat maps, and dendrograms were created using matplotlib (version 3.8.0) and seaborn (version 0.13.0).

### 2.11 Short-chain fatty acids (SCFAs) analysis

A high-performance liquid chromatography (HPLC) Vanquish (Thermo Fisher Scientific, Waltham, Massachusetts, United States) system connected to an RI detector, equipped with an Aminex HPX-87H column 300 mm × 7.8 mm (Bio-Rad), was used to quantify short-chain fatty acids (SCFAs), lactic acid, and ethanol. One millilitre of each sample was mixed with 20 μL of 20% (*v*/*v*) sulphuric acid and incubated for 15 min at 4 °C. The samples were filtered through 0.2 μm pore size PTFE membrane filters and stored at 4 °C. Lactic acid and SCFAs were separated using 5 mM sulphuric acid at a flow rate of 0.5 mL/min, while the column compartment was maintained at 40 °C (28). Standards of lactic acid, acetic acid, propionic acid, and butyric acid in concentrations of 0.1, 1, 2, 5, and 10 mg/mL were prepared in Milli-Q water and analysed under the same conditions as the samples.

## 3 Results

The initial moisture content of the samples was: tarwi, 5.8% ± 0.08; cañihua, 7.4% ± 0.3; and quinoa, 9.28% ± 0.61.

### 3.1 Identification of amino acids and GABA by LC–MS

The identification of amino acids and GABA was based on their derivatization with dansyl chloride, and the molecular weight of the derivatised amino acids was determined using LC–MS. Table 1 shows the molecular weights identified.

**Table 1:** Dasyl-derivatised amino acids detected by LC–MS.

Amino acid	Dansyl amino acid theoretical MW (g/mol)	Retention time (min)	Positive ion polarity (M + H) signal
L-aspartic acid	366.35	7.4	366.83
L-glutamic acid	380.35	8.05	380.9
L-cysteine	473.55	10.43	472.59
L-histidine	388.45	10.63	388.42
L-serine	338.35	10.82	338.7
L-threonine	252.35	11.38	352.74
Glycine	308.32	11.53	308.83
L-alanine	322.34	12.06	322.76
L-arginine	407.45	12.49	407.66
L-proline	348.35	13.53	348.23
L-valine	350.45	15.33	350.69
L-lysine	379.45	15.84	379.41
L-methionine	382.45	16.86	382.1
L-isoleucine	364.45	19.16	364.72
L-leucine	364.45	19.82	362.67
L-phenylalanine	398.45	22.42	398.23

### 3.2 Pretreatment influence on free glutamic acid concentration

**Figure 1:**
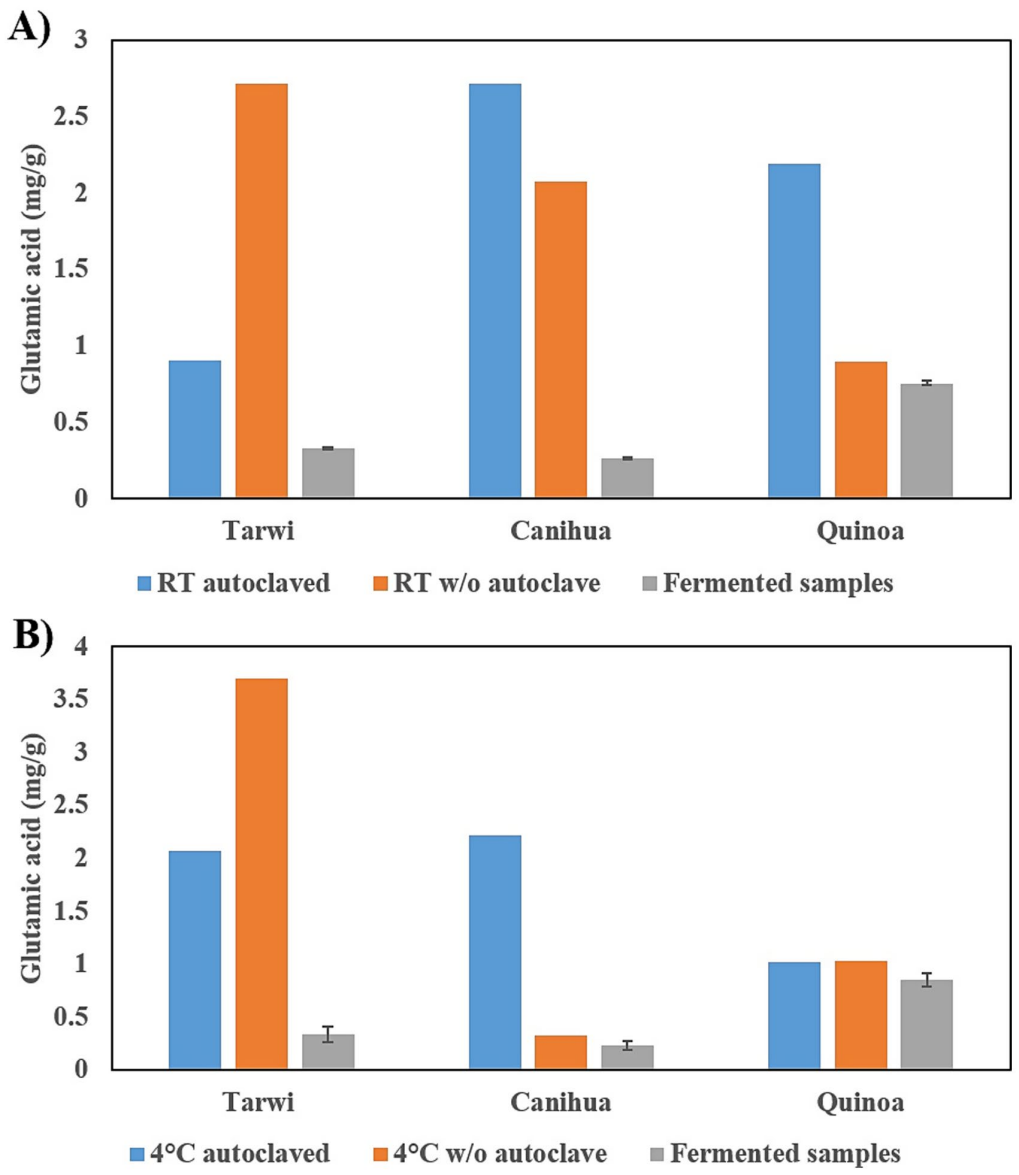
Effect of autoclaving on soluble free glutamic acid content. **(A)** Pretreatment at room temperature, concentration of glutamic acid in mg/g in controls with and without autoclave, and fermented samples. **(B)** Pretreatment at 4 °C, glutamic acid content in controls with and without autoclave and fermented samples. Results from one replicate of controls and duplicates of fermentation, expressed as average ± standard deviation.

Tarwi samples behave differently from cañihua and quinoa when autoclaved (Figure 1). In tarwi seeds, a higher concentration of glutamic acid is observed when the samples are not autoclaved in both RT and 4 °C pretreatments: 2.71 mg/g and 3.69 mg/g, respectively, compared to the autoclaved control samples: 0.9 mg/g at RT and 2.07 mg/g at 4 °C. Indicating a loss of glutamic acid in the autoclaving process. However, cañihua and quinoa exhibit higher concentrations of free glutamic acid after autoclaving.

Comparing the concentration of free glutamic acid with both pretreatments, for tarwi, the pretreatment at 4 °C produces a higher concentration of free glutamic acid, 3.69 mg/g. In contrast, cañihua and quinoa have higher free glutamic acid concentrations when pretreated at RT (around 20 °C) and autoclaved: 0.26 mg/g and 0.75 mg/g, respectively.

**Figure 2:**
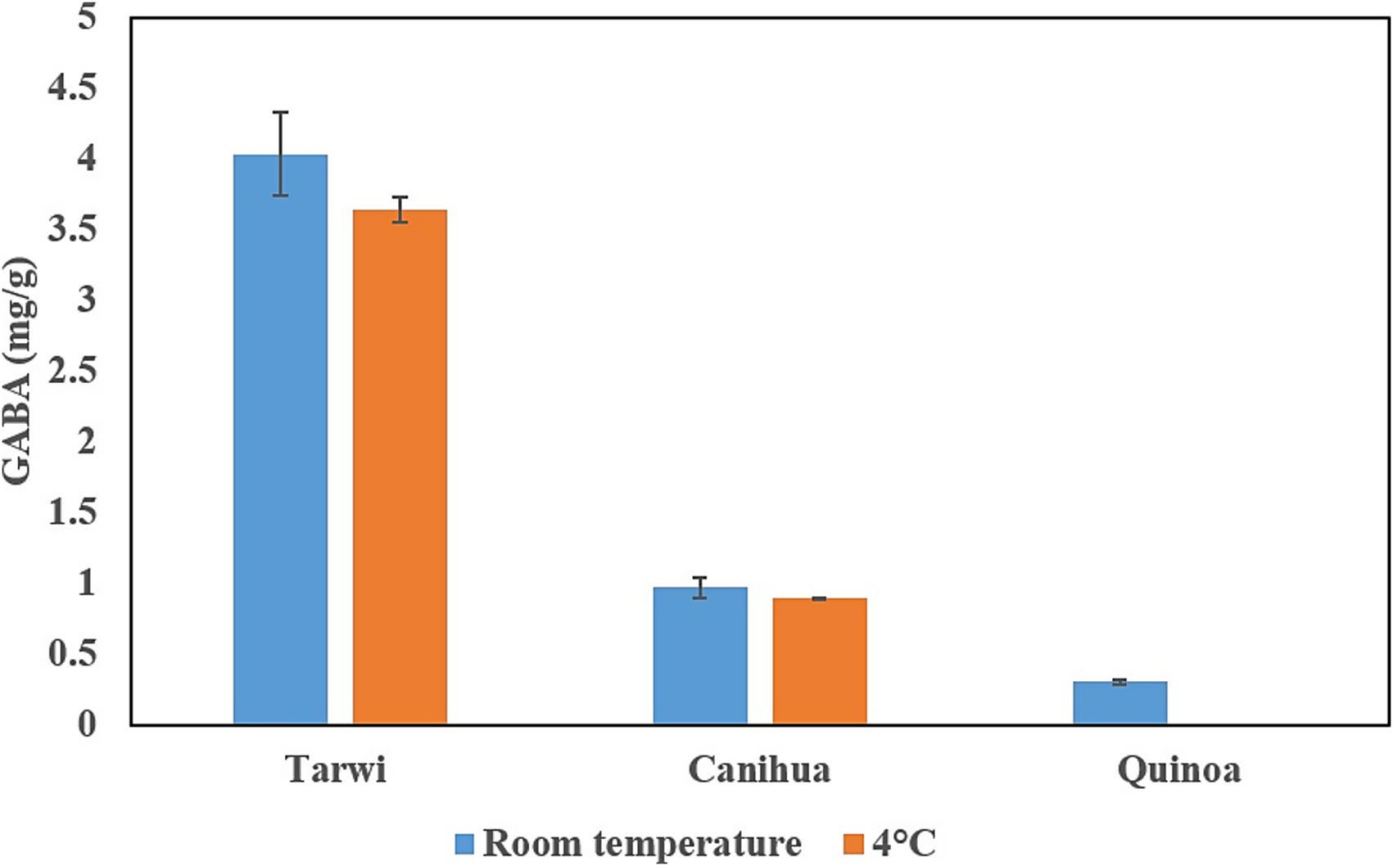
Production of GABA in three Andean seeds by fermentation with *L. brevis*. Two treatments, RT and 4 °C, were compared before the fermentation. Results from duplicates of fermentation.

Finally, after fermentation, the concentration of free glutamic acid decreases in all cases, indicating that glutamic acid could be transformed into GABA; fermented tarwi produced 4.03 mg/g at RT and 3.64 mg/g at 4 °C, fermented cañihua produced 0.97 mg/g at RT and 0.89 mg/g at 4 °C. Finally, quinoa produced 0.3 mg/g of GABA with RT pretreatment, but no GABA was produced in fermented samples pretreated at 4 °C (Figure 2).

### 3.3 Production of GABA by fermentation with *L. brevis*

GABA was produced in all fermentation products except for quinoa samples pretreated at 4 °C. Cohen’s d test shows that GABA production in tarwi and cañihua with room temperature prior treatment has a large effect size (d > 0.8), compared to the 4 °C previous treatment of the seeds (Figure 2).

### 3.4 Change in amino acid profile after fermentation with *L. brevis*

The amino acid profile was determined in all samples, with both prior treatments (RT or at 4 °C).

**Figure 3:**
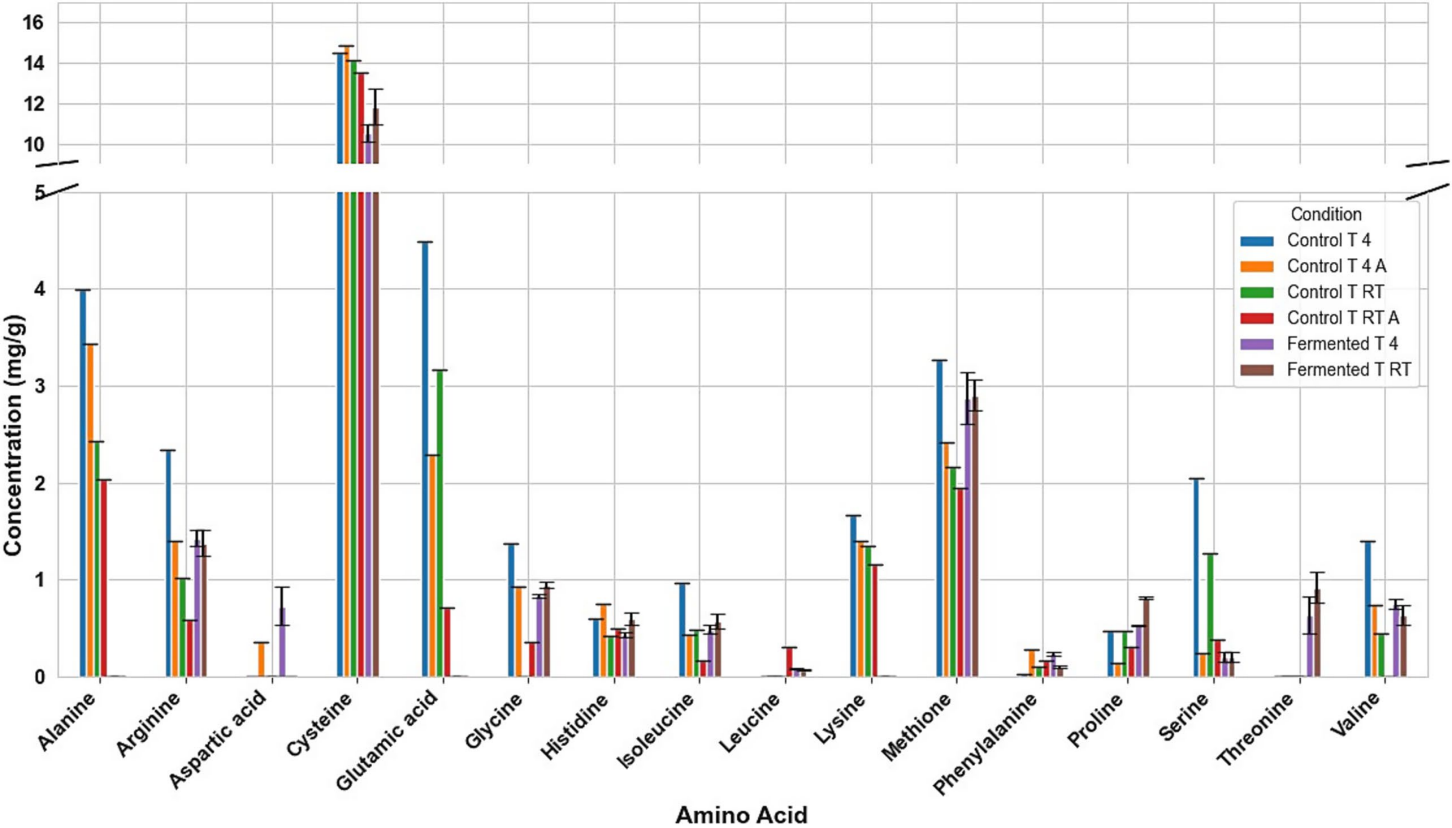
Amino acid profile of Tarwi samples. T RT A, room temperature pretreatment autoclaved; T RT, room temperature pretreatment w/o autoclaving; T 4 A, 4 °C pretreatment autoclaved; T 4, 4 °C pretreatment w/o autoclaving. Results from one replicate of controls and duplicates of fermentation samples, expressed as average ± standard deviation.

**Figure 4:**
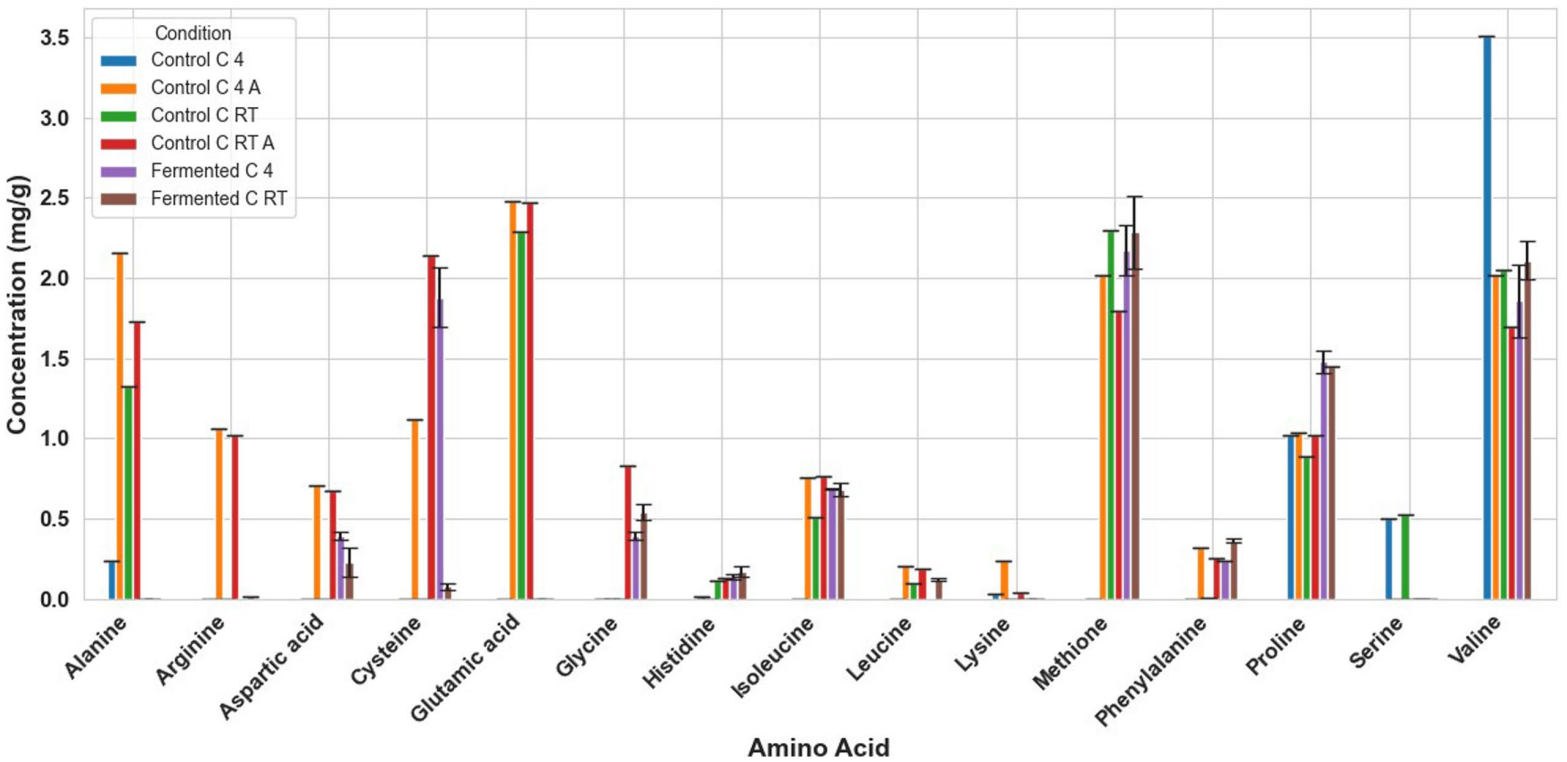
Amino acid profile of cañihua samples. C RT A, room temperature pretreatment autoclaved; C RT, room temperature pretreatment w/o autoclaving; C 4 A, 4 °C pretreatment autoclaved; C 4, 4 °C pretreatment w/o autoclaving. Results from one replicate of controls and duplicates of fermentation samples, expressed as average ± standard deviation.

**Figure 5:**
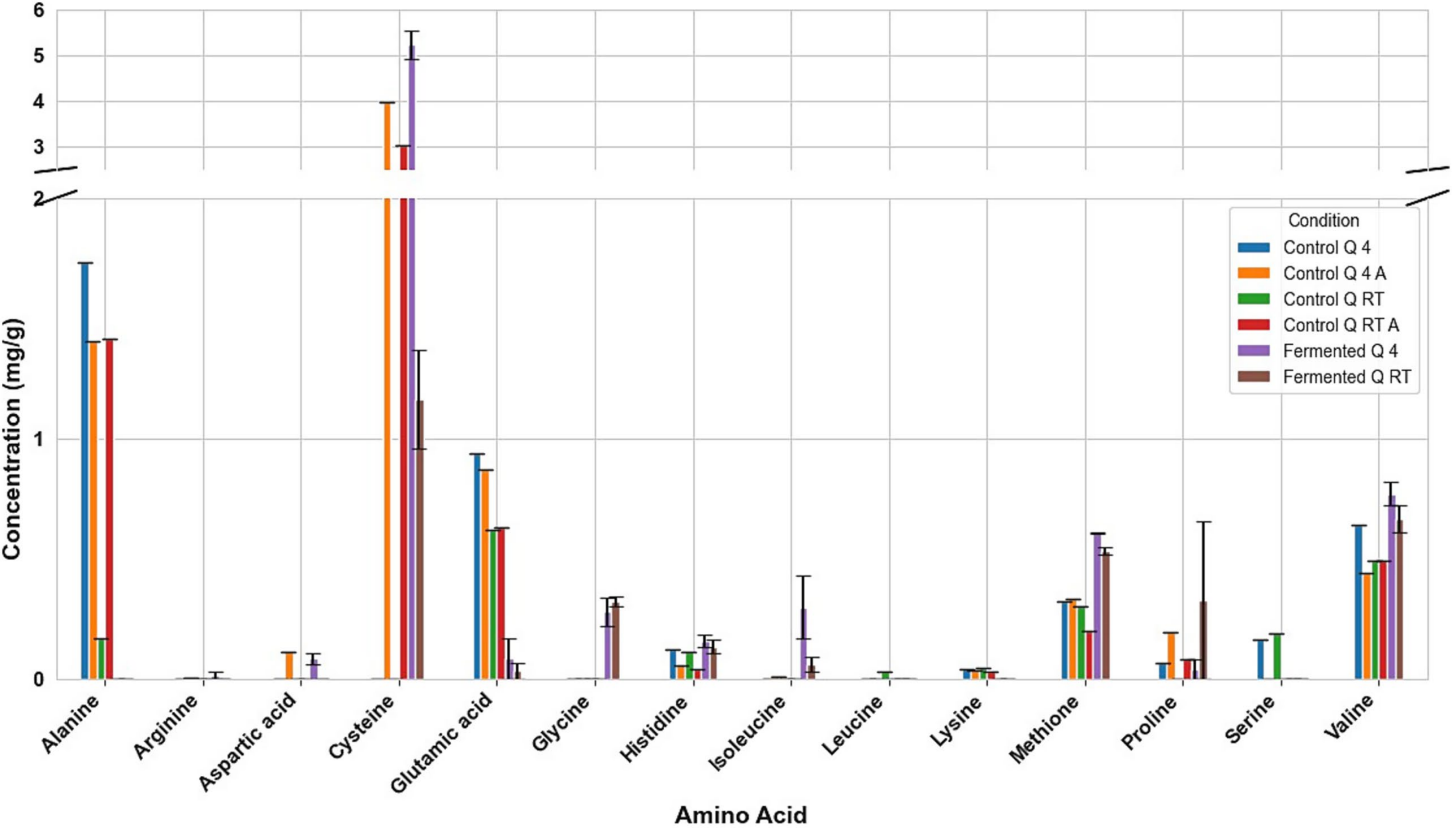
Amino acid profile of quinoa samples. Q RT A, room temperature pretreatment autoclaved; Q RT, room temperature pretreatment w/o autoclaving; Q 4 A, 4 °C pretreatment autoclaved; Q 4, 4 °C pretreatment w/o autoclaving. Results from one replicate of controls and duplicates of fermentation samples, expressed as average ± standard deviation.

Fermentation had different effects on the amino acid composition of every seed. For example, in fermented tarwi, most free amino acids are present in similar amounts regardless of the prior thermal treatment; only a few show differences: cysteine, histidine, threonine, glycine, and proline were higher with prior RT treatment, and aspartic acid and valine were higher in seeds kept at 4 °C before fermentation. The control samples (autoclaved and non-autoclaved) also had the highest concentrations when kept at 4 °C compared to RT (Figure 3). It is also noted that autoclaving decreases the concentration of amino acids compared to non-autoclaved samples. While in cañihua after fermentation, when the seeds were kept at 4 °C previously, aspartic acid, cysteine, and isoleucine were found in higher amounts, while for histidine, glycine, valine, phenylalanine, and leucine, with a previous treatment at RT, were found in higher amounts (Figure 4). For quinoa, alanine is completely consumed during fermentation. Seeds previously treated at 4 °C showed higher amounts of aspartic acid, glutamic acid, cysteine, valine, methionine, and isoleucine. Glycine and proline were higher in seeds kept at RT before fermentation (Figure 5).

The conversion of glutamic acid into GABA was clear. In tarwi, there is almost no glutamic acid after fermentation, and the highest amount of GABA produced by fermentation was 4 mg/g. The same behaviour was observed in cañihua, where almost all glutamic acid was consumed during fermentation, and GABA is produced. On the other hand, a considerable amount of glutamic acid remained in quinoa samples after fermentation, and GABA production was very low. However, no GABA is present in the samples fermented (T 4).

Another interesting result is the influence of autoclaving on the amino acid composition of the seeds. In tarwi seeds, autoclaving resulted in a decrease in the concentration of most amino acids. In cañihua seeds, the opposite effect was observed; most of the amino acids increased in concentration after autoclaving. Finally, in quinoa seeds, there was no clear tendency, unlike in the other seeds; almost half of the amino acids decreased in concentration, while the other half increased in concentration.

**Figure 6:**
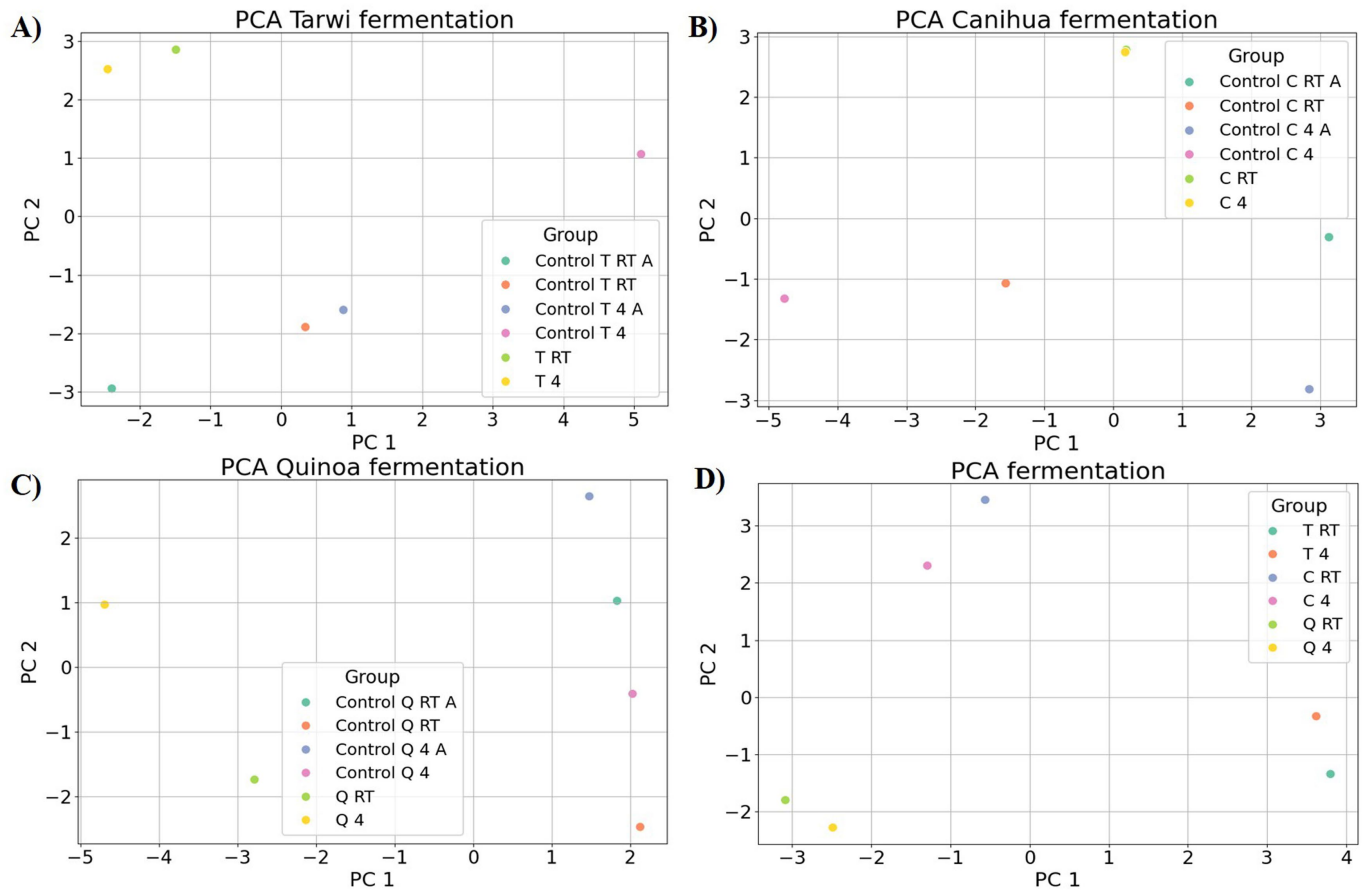
Principal component analysis (PCA) of amino acid profiles across fermentation treatments. **(A)** tarwi, **(B)** cañihua, **(C)** quinoa, and **(D)** all fermented samples. All plots were made in Python (v3.11) using matplotlib (v3.8.0) and seaborn (v0.13.0).

**Figure 7:**
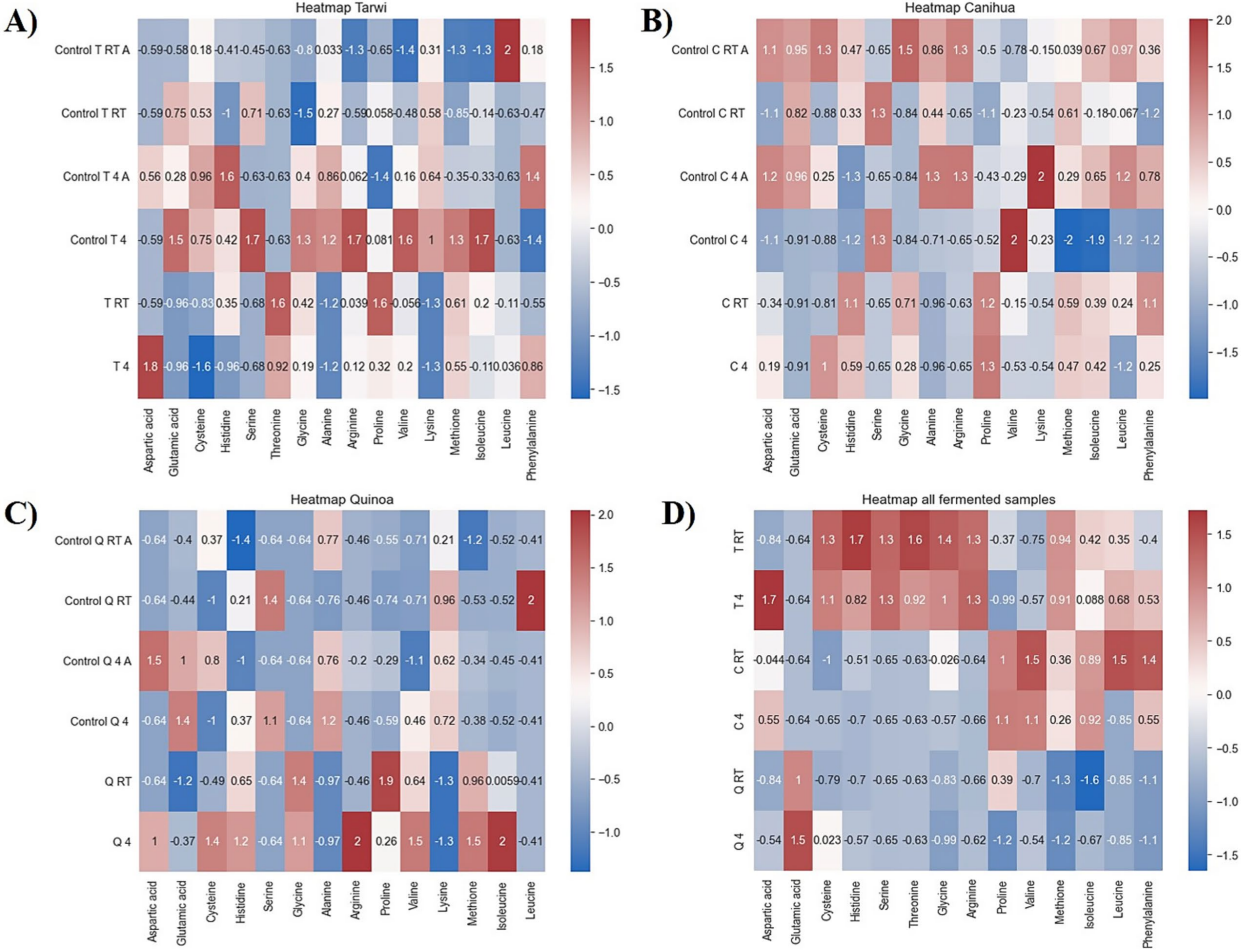
Heat maps of controls. RT, room temperature prior treatment; 4, 4 °C; A, autoclaved control; **(A)** Tarwi, **(B)** Cañihua, **(C)** quinoa, and **(D)** all fermented samples. All plots were made in Python (v3.11) using matplotlib (v3.8.0) and seaborn (v0.13.0).

**Figure 8:**
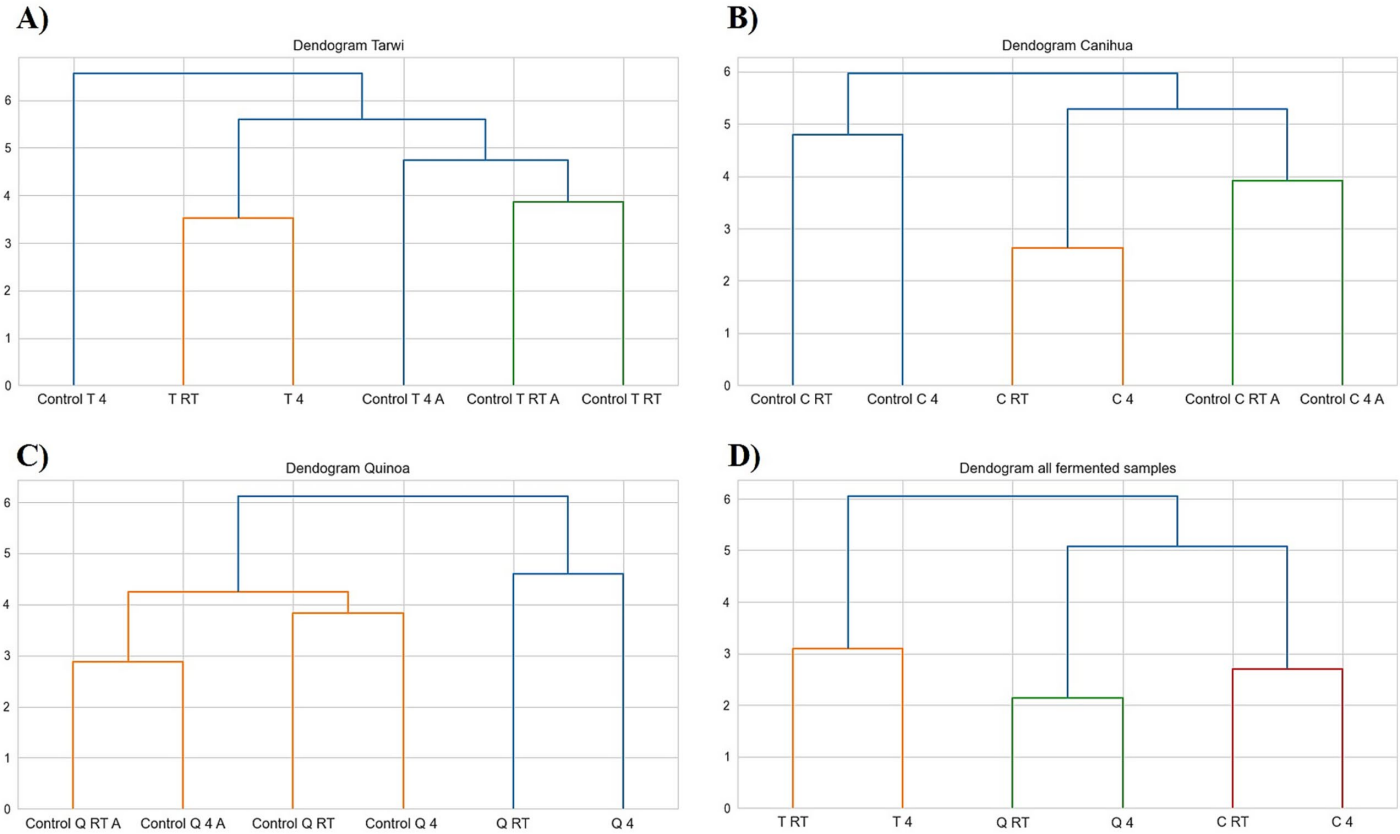
Dendrograms of controls; RT, room temperature prior treatment; 4, 4 °C; A, autoclaved control; **(A)** Tarwi, **(B)** Cañihua, **(C)** Quinoa, and **(D)** all fermented samples. All plots were made in Python (v3.11) using matplotlib (v3.8.0) and seaborn (v0.13.0).

PCA was used to explore the amino acid profiles with prior thermal treatments and fermentation (Figure 6). For tarwi (Figure 6A), PC1 was affected mainly by glutamic acid, serine, alanine, arginine, valine, isoleucine, leucine, and phenylalanine. PC2 captured additional variation, with methionine, threonine, proline, and glycine showing a strong influence, while cysteine and lysine showed a lesser extent. The heat map (Figure 7A) supports that the main increase with prior RT treatment is in threonine and proline, as well as aspartic acid, when the seeds are treated at 4 °C. Fermented samples (T RT and T 4) formed distinct clusters separate from control groups; this was also confirmed in the dendrogram (Figure 8A). The dendrogram also shows that autoclaving affected the controls when a prior 4 °C treatment was applied.

For cañihua, PC1 was primarily influenced by hydrophobic and branched-chain amino acids, including isoleucine, aspartic acid, leucine, valine, and serine. This axis separates samples based on the polarity and hydrophobicity of their amino acids. In the PCA plot (Figure 6B), this separation is evident along PC1, where both controls (RT prior thermal treatment) cluster distinctly from fermented samples, suggesting that fermentation alters the abundance of these amino acids. PC2 was highly influenced by proline, histidine, alanine, and lysine. These amino acids may reflect structural or charge-related shifts during fermentation. PC2 distinguishes both controls (kept at 4 °C) from the other groups, indicating that it may enhance amino acids associated with protein rigidity and aromaticity. These observations are confirmed with the dendrogram (Figure 8B), where autoclaved controls cluster differently from the non-autoclaved ones (independently of the prior treatment). The heat map (Figure 7B) supports that the main increase resulting from fermentation is in proline.

PCA analysis of quinoa fermentation reveals that PC1 is influenced by lysine, alanine, serine, glycine, methionine, and isoleucine. In the PCA plot (Figure 6C), both control groups (RT) cluster at the positive end of PC1, suggesting higher levels of lysine, alanine, and serine. In contrast, both fermented samples shift toward the negative end, indicating fermentation-induced changes in the hydrophobic amino acid content. PC2 is dominated by cysteine, aspartic acid, leucine, and serine. These patterns suggest that PC2 captures variation in sulphur-containing and acidic amino acids. Then, the heat map (Figure 7C) confirms that arginine and isoleucine are the amino acids that increase the most with fermentation after a 4 °C treatment, and proline when the seeds have undergone prior RT treatment. The dendrogram (Figure 8C) suggests that the fermented samples cluster differently from all controls, but the controls are slightly affected by autoclavation, as they form a separate cluster that is still very similar to the non-autoclaved samples.

Finally, the PCA of tarwi, cañihua, and quinoa fermented samples shows that PC1 was primarily driven by glycine, serine, arginine, and threonine, with a high reduction of glutamic acid, reinforcing its conversion into GABA. In the PCA plot (Figure 6D, Table 2), fermented samples kept at RT before fermentation cluster toward the positive end of PC1, showing higher glycine, serine, arginine, and threonine. While fermented samples kept at 4 °C before fermentation shifted leftward, suggesting a reduction in these components over time. PC2 was shaped by valine, phenylalanine, and proline, indicating that this axis captures variation in hydrophobic and aromatic amino acids. Glutamic acid again contributed highly, reinforcing its inverse relationship with fermentation-driven changes. Similarly, PCA (Figure 6D) and the dendrogram (Figure 8D) indicate that all fermented samples cluster differently, depending on the seeds.

**Table 2:** Loadings for PCA analysis for each fermented sample

Amino acid	Tarwi		Cañihua		Quinoa	
	PC1	PC2	PC1	PC2	PC1	PC2
Aspartic acid	−0.13	0.15	0.34	−0.06	−0.11	0.44
Glutamic acid	0.35	−0.13	0.21	−0.32	0.19	0.28
Cysteine	0.26	−0.31	0.26	0.07	−0.19	0.46
Histidine	0.16	−0.01	0.08	0039	−0.26	−0.29
Serine	0.33	−0.03	−0.31	−0.19	0.2	−0.35
Threonine	−0.19	0.37				
Glycine	0.19	0.28	0.2	0.28	−0.35	−0.11
Alanine	0.31	−0.25	0.22	−0.36	0.26	0.29
Arginine	0.3	0.26	0.29	−0.26	−0.28	0.19
Proline	−0.06	0.34	0.04	0.42	−0.27	−0.14
Valine	0.3	0.25	−0.32	−0.13	−0.3	−0.11
Lysine	0.27	−0.31	0.18	−0.32	0.35	0.01
Methionine	0.19	0.38	0.25	0.17	−0.35	−0.05
Isoleucine	0.31	0.25	0.35	0.12	−0.33	0.1
Leucine	−0.23	−0.16	0.3	−0.19	0.13	−0.36
Phenylalanine	−0.2	−0.09	0.29	0.18		

T RT, T4, C RT, C4, Q RT, and Q4.

### 3.5 Production of short-chain fatty acids (SCFAs)

**Figure 9:**
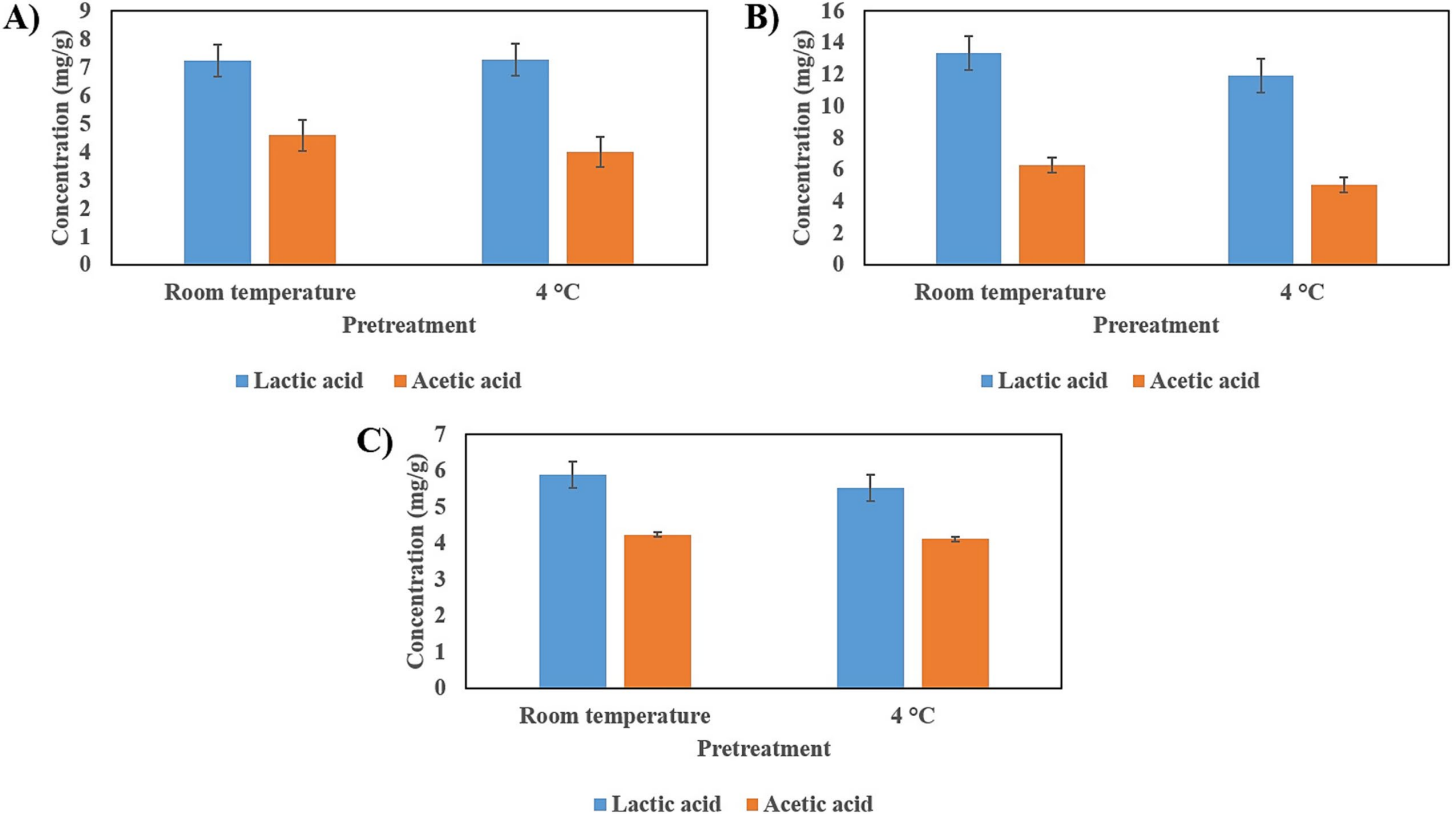
Production of short-chain fatty acids (SCFAs) by fermentation of seeds with *L. brevis*. **(A)** SCFAs produced by fermentation on tarwi seeds. **(B)** SCFAs produced by fermentation on cañihua seeds. **(C)** SCFAs produced by fermentation on quinoa seeds. Results expressed as average ± standard deviation.

In all three seeds, lactic acid and acetic acid were produced. The product of fermentation from cañihua seeds shows the highest levels of those acids (Figure 9).

## 4 Discussion

The production of fermented foods and beverages using LAB has become a significant area of interest for both research and industry, as the metabolites produced can enhance food quality and offer health benefits. One of these metabolites is *γ*-aminobutyric acid (GABA). Many LAB strains have been studied for their capacity to produce GABA, and several authors have concluded that *L. brevis* is the most efficient strain (29). GABA production can vary depending on conditions such as pH, temperature, and the composition of the medium (30).

Fermentation by *L. brevis* of various food matrices to produce GABA has been previously studied. For example, black soybean milk fermented by *L. brevis* FPA 3709 at 37 °C for 48 h achieved a final GABA concentration of 5.42 mg/mL (31). Black raspberry was also fermented by *L. brevis* GABA100 at 37 °C for 15 days, with a final GABA concentration of 2.42 mg/mL (32). Soybean and rice seem to be the most extensively studied food matrices for GABA enrichment (33). Other notable groups that appear to have potential for GABA enrichment include legumes and cereals. Heating and cooling have been tested as pretreatments to see their effect on GABA production by fermentation. Interestingly, on the one hand, a heat drying treatment at 40 °C resulted in increased GABA production in immature soybean seeds, reaching a level of up to 4.48 mg/g (34).

On the other hand, cold stress, if applied to the seeds before fermentation, is efficient in enhancing GABA production in quinoa seeds (21), these authors conclude that the higher GABA produced may be attributed to an adaptive response of the cells to cold, which leads to acidification of the cell membrane, due to the depletion of H^+^ caused by decarboxylation during the synthesis of GABA. However, our results show that GABA production in the three seeds, tarwi, cañihua, and quinoa, with a room temperature treatment of the seeds before fermentation, is higher than that of the prior treatment at 4 °C. However, no heating pretreatments were studied. Recently, in our research group, GABA was produced enzymatically from Andean seeds (26). The production of GABA in tarwi was higher through fermentation compared to the small-scale enzymatic reaction, but lower compared to the large-scale enzymatic reactions (Table 3). Fermented cañihua produced more GABA by enzymatic treatment than fermentation. However, keeping the seeds at 4 °C did show a positive effect, as evidenced by the increased concentration of certain amino acids after fermenting quinoa and canihua seeds. The most significant difference is observed in cysteine concentration after fermentation of both seeds with the pretreatment at 4 °C compared to RT pretreatment. In fermented tarwi seeds, the same effect is observed in almost all amino acids, but not for threonine, proline, and isoleucine.

**Table 3:** Comparison of enzymatic and fermentation production of GABA in Andean seeds with *L. brevis.*

Seed	GABA mg/g of ground seed	Process to obtain GABA/conditions	Scale (volume)	Reference
Tarwi	2.46	Enzymatic conversion by *L. brevis* GadB at 37 °C, pH 7 for 14 h.	1 mL	(26)
	14.22	Enzymatic conversion by *L. brevis* GadA at 37 °C, pH 7 for 14 h.	100 mL	(26)
	4.03	Fermentation with *L. brevis* for 48 h at 30 °C. With room temperature pretreatment.	25 mL	Present work
Cañihua	0.27	Enzymatic conversion by *L. brevis* GadA at 37 °C, pH 7 for 14 h.	1 mL	(26)
	0.97	Fermentation with *L. brevis* for 48 h at 30 °C. With room temperature pretreatment.	25 mL	Present work
Quinoa	0.41	Enzymatic conversion by *L. brevis* GadB at 37 °C, pH 7 for 14 h. Pretreatment with pancreatin.	1 mL	(26)
	0.3	Fermentation with *L. brevis* for 48 h at 30 °C. With room temperature pretreatment.	25 mL	Present work

Enzymatic production was previously performed in the group, and fermentation was conducted in the present study. Both studies used the same seed samples.

Short-chain fatty acids (SCFAs) and lactic acid are also important products of LAB fermentation. In this study, only lactic and acetic acid were produced through fermentation by *L. brevis,* with higher concentrations of lactic acid than acetic acid being observed. Other studies have identified a group of sugars that, when present in the culture medium, consistently produce more lactic than acetic acid, regardless of the *L. brevis* strain (35). These sugars are D-glucose, D-galactose, D-mannose, and D-mannitol, suggesting that some of these sugars may be present in the seeds used in this study, which could lead to a higher production of lactic acid than acetic acid in all cases. Acetic acid has been found to enhance the production of anti-inflammatory mediators, including IL-10, transforming growth factor-β (TGF-β), and annexin A1 (36). Lactic acid has demonstrated various bioactive capacities, including its critical role in modulating inflammation in a model of small intestine injury caused by indomethacin (37). It has also been shown to contribute to maintaining intestinal barrier function (38).

Little is known about the effect of autoclaving on the free amino acid profile before fermentation, especially in Andean seeds. Early studies reported a 10% decrease in lysine after autoclaving chickpea and pulses but no change in other amino acids (39). Another study also found a decline in lysine when autoclaving blends of soybean, white beans, sesame, and peanut (40). However, recent work found no change in the amino acid profile when cheese whey permeates are autoclaved (41). In the present work, autoclaving affected the amino acid profiles of tarwi, cañihua, and quinoa in distinct ways.

Some free amino acids have increased in the soluble phase after the fermentation of the Andean seeds. Tarwi’s fermentation product shows that threonine was produced. This essential amino acid is a crucial component of gastrointestinal mucin and serves as a nutritional modulator that influences the intestinal immune system through complex signalling networks (42). Other essential amino acids increased in concentration after the fermentation of tarwi by *L. brevis*, including histidine, isoleucine, leucine, methionine, and valine. In fermented cañihua, glycine is not observed in the controls of soluble amino acids but is present after fermentation. This non-essential amino acid has also been suggested as a conditionally essential amino acid, as it has been observed that glycine supplementation is needed in metabolic disorders such as obesity and type 2 diabetes (43). Glycine is not only a building block of proteins, but it is also required for many metabolic processes, and it has a broad spectrum of anti-inflammatory, cytoprotective, and immunomodulatory properties (44). Other essential amino acids that increased in concentration after the fermentation of cañihua by *L. brevis* are histidine, phenylalanine, isoleucine, and methionine. In fermented quinoa, glycine and isoleucine are produced during the fermentation. Isoleucine is an essential amino acid and together with leucine and valine it comprises 17% of the human skeletal muscle and is used to support protein synthesis (45). Methionine, histidine, and valine are other essential amino acids that are increased in concentration by quinoa fermentation.

Besides the beneficial effects of free amino acids, they can also influence the taste of fermented food. They have been classified into four groups: amino acids that contribute umami taste (aspartic acid and glutamic acid), amino acids that contribute to sweetness (serine, alanine, glycine and threonine), amino acids that contribute to bitterness (arginine, histidine, tyrosine, leucine, valine, methionine, isoleucine, phenylalanine, lysine and proline), and finally cysteine is considered tasteless (46). In this sense, fermented tarwi exhibits an increase in the concentration of free amino acids that contribute to bitter taste, but only one that could contribute to its sweet taste. Fermented cañihua and quinoa have an increase in concentration of two bitter-contributor amino acids and one sweet-contributor. It can be concluded that the amino acid profile in fermented products may contribute to an increased bitterness.

In this exploratory study, a limitation is that fermentations were conducted in duplicate due to restricted seed availability and the high analytical demand of LC–MS/HPLC analyses. Nevertheless, all analytical measurements were performed in duplicate for each replicate, and results were consistent, indicating good reproducibility. Future work will include larger-scale fermentations with additional replicates to strengthen statistical robustness. In addition, the study of organoleptic properties of fermented Andean seeds aims to develop nutritional and functional fermented foods, extend their shelf life, and enhance their palatability.

## 5 Conclusion

This study explores the enhancement of the functional value of Andean seeds through fermentation with *L. brevis* DSM 1269. As an environmentally friendly technique, it enables the production of bioactive compounds such as lactic acid, acetic acid, and GABA, while also increasing the production of essential amino acids, including threonine, histidine, methionine, isoleucine, leucine, valine, and lysine. In this sense, these results could lead to scaling up the process and potentially applying it to formulate novel, fermented, plant-based foods with enhanced properties.

## Data Availability

The raw data supporting the conclusions of this article will be made available by the authors, without undue reservation.

## References

[1] Gan R-Y Li H-B Gunaratne A Sui Z-Q Corke H. Effects of fermented edible seeds and their products on human health: bioactive components and bioactivities. Compr Rev Food Sci Food Saf. (2017) 16:489–531. doi: 10.1111/1541-4337.12257, PMID: 33371560

[2] Terefe NS. 5 - recent developments in fermentation technology: toward the next revolution in food production In: Juliano P Buckow R Nguyen MH Knoerzer K Sellahewa J, editors. Food engineering innovations across the food supply chain. Amsterdam, Netherlands: Elsevier (2022).

[3] Abdul Hakim BN Xuan NJ Oslan SNH. A comprehensive review of bioactive compounds from lactic acid bacteria: potential functions as functional food in dietetics and the food industry. Foods. (2023) 12:2850. doi: 10.3390/foods12152850, PMID: 37569118 PMC10417365

[4] Lu Y Tan X Lv Y Yang G Chi Y He Q. Physicochemical properties and microbial community dynamics during Chinese horse bean-chili-paste fermentation, revealed by culture-dependent and culture-independent approaches. Food Microbiol. (2020) 85:103309. doi: 10.1016/j.fm.2019.103309, PMID: 31500715

[5] Yu J Geng Y Xia H Ma D Liu C Wu R . Lab fermentation improves production of bioactive compounds and antioxidant activity of *Withania somnifera* extract and its metabolic signatures as revealed by LC-MS/MS. J Microbiol Biotechnol. (2022) 32:473–83. doi: 10.4014/jmb.2111.11018, PMID: 35058401 PMC9628816

[6] Nkhata SG Ayua E Kamau EH Shingiro JB. Fermentation and germination improve nutritional value of cereals and legumes through activation of endogenous enzymes. Food Sci Nutr. (2018) 6:2446–58. doi: 10.1002/fsn3.846, PMID: 30510746 PMC6261201

[7] Pranoto Y Anggrahini S Efendi Z. Effect of natural and *Lactobacillus plantarum* fermentation on in-vitro protein and starch digestibilities of sorghum flour. Food Biosci. (2013) 2:46–52. doi: 10.1016/j.fbio.2013.04.001

[8] Jin Y Wu J Hu D Li J Zhu W Yuan L . Gamma-aminobutyric acid-producing Levilactobacillus brevis strains as probiotics in litchi juice fermentation. Foods. (2023) 12:302. doi: 10.3390/foods12020302, PMID: 36673393 PMC9857889

[9] Ngo D-H Vo TS. An updated review on pharmaceutical properties of gamma-aminobutyric acid. Molecules. (2019) 24:2678. doi: 10.3390/molecules24152678, PMID: 31344785 PMC6696076

[10] Bruni O Ferini-Strambi L Giacomoni E Pellegrino P. Herbal remedies and their possible effect on the GABAergic system and sleep. Nutrients. (2021) 13:530. doi: 10.3390/nu13020530, PMID: 33561990 PMC7914492

[11] Hepsomali P Groeger JA Nishihira J Scholey A. Effects of oral gamma-aminobutyric acid (GABA) administration on stress and sleep in humans: a systematic review. Front Neurosci. (2020) 14:923. doi: 10.3389/fnins.2020.00923, PMID: 33041752 PMC7527439

[12] Jeong A-H Hwang J Jo K Kim S Ahn Y Suh HJ . Fermented gamma-aminobutyric acid improves sleep behaviors in fruit flies and rodent models. Int J Mol Sci. (2021) 22:3537. doi: 10.3390/ijms22073537, PMID: 33805468 PMC8036604

[13] Wu S-J Chang C-Y Lai Y-T Shyu Y-T. Increasing γ-aminobutyric acid content in vegetable soybeans via high-pressure processing and efficacy of their antidepressant-like activity in mice. Foods. (2020) 9:1673. doi: 10.3390/foods9111673, PMID: 33207592 PMC7696959

[14] Quillin SJ Tran P Prindle A. Potential roles for gamma-aminobutyric acid signaling in bacterial communities. Bioelectricity. (2021) 3:120–5. doi: 10.1089/bioe.2021.0012, PMID: 34476387 PMC8380936

[15] Dhakal R Bajpai VK Baek KH. Production of gaba (γ-aminobutyric acid) by microorganisms: a review. Braz J Microbiol. (2012) 43:1230–41. doi: 10.1590/s1517-83822012000400001, PMID: 24031948 PMC3769009

[16] Zhang Y Zhu M Lu W Zhang C Chen D Shah NP . Optimizing Levilactobacillus brevis NPS-QW 145 fermentation for gamma-aminobutyric acid (GABA) production in soybean sprout yogurt-like product. Foods. (2023) 12:977. doi: 10.3390/foods12050977, PMID: 36900494 PMC10000865

[17] Cataldo PG Urquiza Martínez MP Villena J Kitazawa H Saavedra L Hebert EM. Comprehensive characterization of γ-aminobutyric acid (GABA) production by Levilactobacillus brevis CRL 2013: insights from physiology, genomics, and proteomics. Front Microbiol. (2024) 15:1408624. doi: 10.3389/fmicb.2024.1408624, PMID: 38962125 PMC11219586

[18] Luo X Wang Y Li Q Wang D Xing C Zhang L . Accumulating mechanism of γ-aminobutyric acid in soybean (*Glycine max* L.) during germination. Int J Food Sci Technol. (2018) 53:106–11. doi: 10.1111/ijfs.13563

[19] Gomez Cahuata JF Rosas-Quina YE Pachari Vera E. Cañihua (*Chenopodium pallidicaule* Aellen) a promising superfood in food industry: a review. Nutr Food Sci. (2022) 52:917–28. doi: 10.1108/NFS-09-2021-0277

[20] Suomela J-P Repo-Carrasco-Valencia R Lutz M. Andean native grains, quinoa, and lupin as sources of bioactive components In: Native crops in Latin America. Boca Raton, Florida, USA: CRC Press (2022).

[21] Zhang Y Zhang M Li T Zhang X Wang L. Enhance production of γ-aminobutyric acid (GABA) and improve the function of fermented quinoa by cold stress. Foods. (2022) 11:3908. doi: 10.3390/foods11233908, PMID: 36496716 PMC9737818

[22] De-La-Cruz-Yoshiura S Vidaurre-Ruiz J Alcázar-Alay S Encina-Zelada CR Cabezas DM Correa MJ . Sprouted Andean grains: an alternative for the development of nutritious and functional products. Food Rev Int. (2023) 39:5583–611. doi: 10.1080/87559129.2022.2083158

[23] Verduzco-Oliva R Gutierrez-Uribe JA. Beyond enzyme production: solid state fermentation (SSF) as an alternative approach to produce antioxidant polysaccharides. Sustainability. (2020) 12:495. doi: 10.3390/su12020495

[24] Ghosh JS. Solid state fermentation and food processing: a short review. J Nutr Food Sci. (2016) 6:1–7. doi: 10.4172/2155-9600.1000453

[25] Karabulut G Nemzer BV Feng H. γ-Aminobutyric acid (GABA)-enriched hemp milk by solid-state co-fermentation and germination bioprocesses. Plant Foods Hum Nutr. (2024) 79:322–9. doi: 10.1007/s11130-024-01187-6, PMID: 38753215 PMC11178579

[26] Ibieta G Ortiz-Sempértegui J Peñarrieta JM Linares-Pastén JA. Enhancing the functional value of Andean food plants: enzymatic production of γ-aminobutyric acid from tarwi, cañihua and quinoa real seeds’ proteins. LWT Food Sci Technol. (2025) 220:117564. doi: 10.1016/j.lwt.2025.117564

[27] Villegas JM Brown L Savoy de Giori G Hebert EM. Optimization of batch culture conditions for GABA production by *Lactobacillus brevis* CRL 1942, isolated from quinoa sourdough. LWT Food Sci Technol. (2016) 67:22–6. doi: 10.1016/j.lwt.2015.11.027

[28] Allahgholi L Jönsson M Christensen MD Jasilionis A Nouri M Lavasani S . Fermentation of the brown seaweed *Alaria esculenta* by a lactic acid bacteria consortium able to utilize mannitol and laminari-oligosaccharides. Fermentation. (2023) 9:499. doi: 10.3390/fermentation9060499

[29] Wang Q Liu X Fu J Wang S Chen Y Chang K . Substrate sustained release-based high efficacy biosynthesis of GABA by *Lactobacillus brevis* NCL912. Microb Cell Factories. (2018) 17:1–8. doi: 10.1186/s12934-018-0919-6 PMC5960080 29778094

[30] Icer MA Sarikaya B Kocyigit E Atabilen B Çelik MN Capasso R . Contributions of gamma-aminobutyric acid (GABA) produced by lactic acid bacteria on food quality and human health: current applications and future prospects. Foods. (2024) 13:2437. doi: 10.3390/foods13152437, PMID: 39123629 PMC11311711

[31] Ko CY Lin H-TV Tsai GJ. Gamma-aminobutyric acid production in black soybean milk by *Lactobacillus brevis* FPA 3709 and the antidepressant effect of the fermented product on a forced swimming rat model. Process Biochem. (2013) 48:559–68. doi: 10.1016/j.procbio.2013.02.021

[32] Kim JY Lee MY Ji GE Lee YS Hwang KT. Production of γ-aminobutyric acid in black raspberry juice during fermentation by *Lactobacillus brevis* GABA100. Int J Food Microbiol. (2009) 130:12–6. doi: 10.1016/j.ijfoodmicro.2008.12.028, PMID: 19167126

[33] Lee XY Tan JS Cheng LH. Gamma-aminobutyric acid (GABA) enrichment in plant-based food – a mini review. Food Rev Int. (2023) 39:5864–85. doi: 10.1080/87559129.2022.2097257

[34] Takahashi Y Sasanuma T Abe T. Accumulation of gamma-aminobutyrate (GABA) caused by heat-drying and expression of related genes in immature vegetable soybean (edamame). Breed Sci. (2013) 63:205–10. doi: 10.1270/jsbbs.63.205, PMID: 23853515 PMC3688382

[35] Hwang HJ Lee SY Kim SM Lee SB. Fermentation of seaweed sugars by *Lactobacillus* species and the potential of seaweed as a biomass feedstock. Biotechnol Bioprocess Eng. (2011) 16:1231–9. doi: 10.1007/s12257-011-0278-1

[36] Vieira AT Galvão I Macia LM Sernaglia EM Vinolo MAR Garcia CC . Dietary fiber and the short-chain fatty acid acetate promote resolution of neutrophilic inflammation in a model of gout in mice. J Leukoc Biol. (2017) 101:275–84. doi: 10.1189/jlb.3A1015-453RRR 27496979

[37] Watanabe T Nishio H Tanigawa T Yamagami H Okazaki H Watanabe K . Probiotic *Lactobacillus casei* strain Shirota prevents indomethacin-induced small intestinal injury: involvement of lactic acid. American journal of physiology-gastrointestinal and liver. Physiology. (2009) 297:G506–13. doi: 10.1152/ajpgi.90553.2008 19589943

[38] Garrote GL Abraham AG Rumbo M. Is lactate an undervalued functional component of fermented food products? Front Microbiol. (2015) 6:629. doi: 10.3389/fmicb.2015.00629, PMID: 26150815 PMC4473639

[39] del Cueto AG Martinez W Frampton V. Heat effects on peas, effect of autoclaving on the basic amino acids and proteins of the chick pea. J Agric Food Chem. (1960) 8:331–2. doi: 10.1021/jf60110a022

[40] Srihara P Alexander J. Protein quality of raw and autoclaved plant protein blends. Can Inst Food Sci Technol J. (1983) 16:63–7. doi: 10.1016/S0315-5463(83)72021-3

[41] Donzella S Fumagalli A Arioli S Pellegrino L D’Incecco P Molinari F . Recycling food waste and saving water: optimization of the fermentation processes from cheese whey permeate to yeast oil. Fermentation. (2022) 8:341. doi: 10.3390/fermentation8070341

[42] Tang Q Tan P Ma N Ma X. Physiological functions of threonine in animals: beyond nutrition metabolism. Nutrients. (2021) 13:2592. doi: 10.3390/nu13082592, PMID: 34444752 PMC8399342

[43] Alves A Bassot A Bulteau A-L Pirola L Morio B. Glycine metabolism and its alterations in obesity and metabolic diseases. Nutrients. (2019) 11:1356. doi: 10.3390/nu11061356, PMID: 31208147 PMC6627940

[44] Pérez-Torres I María Zuniga-Munoz A Guarner-Lans V. Beneficial effects of the amino acid glycine. Mini Rev Med Chem. (2017) 17:15–32. doi: 10.2174/1389557516666160609081602 27292783

[45] Gorissen SHM Phillips SM. Chapter 17 - branched-chain amino acids (leucine, isoleucine, and valine) and skeletal muscle In: Walrand S, editor. Nutrition and skeletal muscle. San Diego, California: Academic Press (2019).

[46] Kim Y Kim E-Y Son HJ Lee J-j Choi Y-h Rhyu M-R. Identification of a key umami-active fraction in modernized Korean soy sauce and the impact thereof on bitter-masking. Food Chem. (2017) 233:256–62. doi: 10.1016/j.foodchem.2017.04.123, PMID: 28530573

